# Epigenome-wide association study reveals CpG sites related to COG of neuroblastoma

**DOI:** 10.1042/BSR20200826

**Published:** 2020-05-28

**Authors:** Hao Zhao, Xiaojun Zhou, Hu Sun, Dongyun Zhao, Hongfei Liu, Bin Huang, Xingang Li, Yinghao Gu

**Affiliations:** 1 Department of Neurosurgery, Qilu Hospital of Shandong University and Institute of Brain and Brain-Inspired Science, Shandong University, Jinan 250012, Shandong, China; 2 Department of Neurosurgery, Zibo Central Hospital Affiliated to Shandong University, Zibo 255036, Shandong, China; 3 Department of Paediatric Neurology, Zibo Central Hospital Affiliated to Shandong University, Zibo 255036, Shandong, China; 4 Shandong Key Laboratory of Brain Function Remodeling, Qilu Hospital of Shandong University, Jinan 250012, Shandong, China

**Keywords:** CpG, EWAS, Logistic regression model, Neuroblastoma, Risk classification

## Abstract

**Background.** Neuroblastoma (NB) is the most common extracranial solid tumor in infants and children. Its variable location and complex pathogenesis make NB hard for early diagnosis and risk classification. **Methodology.** We analyzed the methylation data of 236 samples from patients with NB in Therapeutically Applicable Research to Generate Effective Treatments (TARGET) database. Kaplan–Meier survival analysis was used for comparing overall survival of NB patients in different groups. Epigenome-wide association study (EWAS) was conducted to screen CpGs significantly associated with NB patients’ Children’s Oncology Group (COG). Logistic regression method was used for constructing a model to predict NB patients’ COG. **Results.** NB patients in low COG showed significantly superior prognosis than those in high COG. A total of seven CpG sites were found closely related to COG. Logistic regression model based on those CpGs showed superior performance in separating NB patients in different COGs. **Conclusions.** The present study highlights the important role of DNA methylation in NB development, which might provide evidence for treatment decisions for children NB.

## Introduction

Neuroblastoma (NB) is one of the most common malignant tumors in infants and children. The incidence of NB in extracranial solid tumor in childhood. Approximately 7% of the malignant tumors occur in children born to 14 years old, with a fatality rate of up to 15% [1]. NB usually originates from the sympathetic nervous system in abdomen or chest, most commonly from adrenal gland. Patients with different origins and spread of tumor cells exhibit different symptoms, such as abdominal distension, constipation, dyspnea, skin mass, bone pain and anemia [2]. NB is a self-limiting disease, which is possible for spontaneously cured and recovery when the age of diagnosis is less than 18 months [3]. Children with NB are usually treated with surgical resection, chemotherapy, radiotherapy, and autologous hematopoietic stem cell transplantation. Appropriate treatments based on risk classification could reduce the treatment-related toxicities and improve the prognosis. Patients with low or intermediate risk have satisfactory prognosis, while the recurrence rate of patients with high risk is more than 50%, with 5-year survival rate of 40–50% [4]. However, the occult primary site and diverse phenotypes make NB hard for early diagnosis and most of the patients were at high risk when diagnosed. Advanced NB is highly invasive and rapidly progressing, leading to difficulty in treatment and poor prognosis [5]. Staging and risk classification are two important prognostic factors of NB. The International Neuroblastoma staging system (INSS), which is the most widely used staging standard, classifies NB tumors into stage I, II, III, IV and IV-S according to the tumor resectability [6]. The biological and clinical characteristics of NB are complex, which would be difficult to evaluate the development and risk before treatment only by relying on INSS. Therefore, the International Neuroblastoma Risk Group (INRG) proposed a new stage and risk classification system for pretreatment. According to INSS stage, age of diagnosis, MYCN amplification status, DNA ploidy, and histopathology, patients with NB are classified into low-risk group, intermediate-risk group, and high-risk group [7]. Children’s Oncology Group (COG) suggests that the therapy strategy should be made according to the risk classification of NB [8,9]

Assessment of NB COG risk classification requires a lot of comprehensive information, and subjective factors of medical staff are involved. To reduce the uncertain subjectivity and improve the accuracy of risk classification, we performed epigenome-wide association study (EWAS) on methylation data of patients with NB to identify methylation variations that are related to NB COG risk classification. In addition, a logistic regression model was constructed using these CpG sites for accurate prediction of patients’ COG.

## Materials and methods

### Methylation data of NB

The methylation data of NB were obtained from Therapeutically Applicable Research to Generate Effective Treatments (TARGET) database (https://ocg.cancer.gov/programs/target), which contains information of common tumors in children. We obtained the methylation data and corresponding clinical data of 236 NB patients from TARGET database. All 236 samples were tumor samples, of which 169 samples contained complete survival information. Detailed epidemiological features of those 169 samples are provided in Table 1. The methylation data were detected by Illumina Human Methylation 450 (HM450) arrays platform.

**Table 1:** Clinicopathological characteristics of NBL patients from TARGET database

Characteristics		NBL patients (*n*=169)	
		No.	%
**Sex**	Female	66	39.05%
	Male	103	60.95%
**Race**	White	123	72.78%
	Black or African American	28	16.57%
	Asian	1	0.59%
	Native Hawaiian or other Pacific Islander	3	1.78%
	Unknown	15	8.88%
**Pathologic stage**	I	7	4.14%
	II	1	0.59%
	III	5	2.96%
	IV	156	92.31%
**Survival time**	Long (>5 years)	87	51.48%
	Short (<5 years)	82	48.52%
**Survival status**	Dead	79	46.75%
	Alive	90	53.25%

### EWAS

Similar to genome-wide association study (GWAS), which finds genetic mutations in the whole-genome by comparing case group and control group, EWAS is to identify methylation variations that are related to disease by comparing different sample groups at epigenetic level. We used CpGassoc package (version 2.60, https://CRAN.R-project.org/package=CpGassoc) of R language to study the relationship between methylation level and phenotype. In this package, the matrix of methylation β value and the sample phenotype information, here refers to COG risk group, were used as input, and the *P*-value that represented the relationship between each CpG site and phenotype was calculated by chi-square test. The smaller the *P*-value is, the closer the relationship between the CpG site and phenotype is. *P*-value < 1e-8 was used as the threshold for the significant association between CpG site and COG risk group.

### Construction of logistic regression model

As a common approach in classification, logistic regression is based on a set of variables to predict the classification results. In the present study, the β value of methylation site is used to predict samples’ COG. The 236 samples were randomly divided into training set and testing set with the same sample size. Based on the β value of CpG site that screened in the previous analysis in the training set, the logistic regression model was constructed with R language. Testing set was used to evaluate the accuracy of the regression model.

### Statistical analysis

For the 169 samples with survival information, we used the survival package (version 2.42, https://www.rdocumentation.org/packages/survival) of R language (version 3.5, https://www.r-project.org/) to perform Kaplan–Meier survival analysis. Cox proportional hazards model, which introduced mitosis-karyorrhexis index (MKI), risk groups (COG_risk_group), and tumor percentage (percent_tumor) as variables, was used to calculate the hazard rate (HR) between the sample groups based on each variable. Fisher’s exact test was used to determine the significance of the overall survival between different sample groups.

## Results

### MKI and COG risk group significantly affect the prognosis and survival

**Figure 1:**
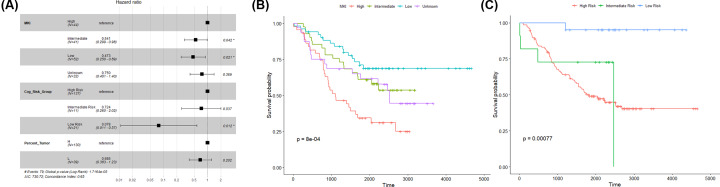
Correlation between different variables and prognosis (**A**) Hazard ratio of sample groups based on MKI, Cog_risk_group, and percent_tumor, respectively. (*n*, number of samples; *P-value <0.05). (**B**) Kaplan–Meier survival curves of the correlation between MKI and survival probability. (**C**) Kaplan–Meier survival curves of the correlation between COG_risk_group and survival probability.

Hazard ratio was calculated based on the Cox proportional hazards model. All 169 samples were divided into groups according to MKI (high, intermediate, low, and unknown), COG risk group (high risk, intermediate risk, and low risk), and tumor percentage (H: > 50%, L: < 50%), respectively. HR > 1 indicated that samples with the factor had a higher risk of death than reference (high MKI, low COG risk group, or low tumor percentage), and HR < 1 indicated samples with the factor had a lower risk of death than reference. Lower.95 and upper.95 are 95% confidence intervals, and *P*-value<0.05 was considered as threshold. Figure 1A showed that NB patients with low and intermediate MKI had lower risk of death compared with high MKI. Compared with high COG, low and intermediate COG samples had lower risk of death. Tumor percentage showed no significant correlation with prognosis. Kaplan–Meier survival analysis results also supported that low MKI (Figure 1B) and low COG (Figure 1C) correlated with superior NB prognosis.

### EWAS analysis

**Figure 2:**
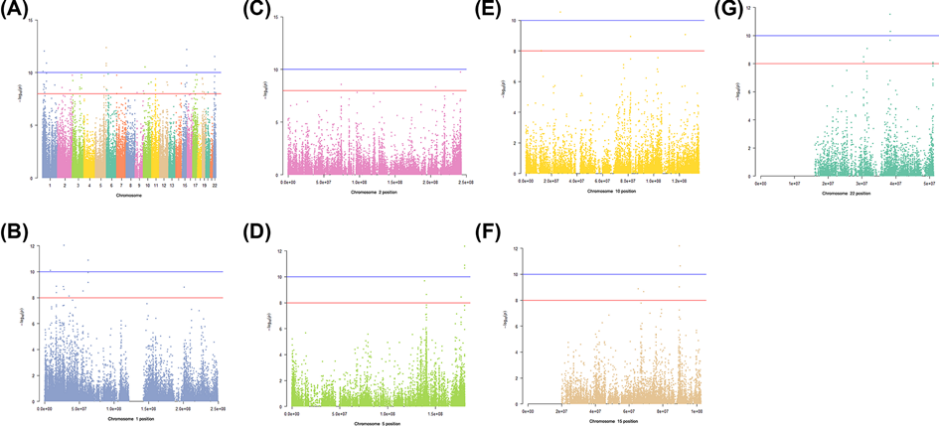
Manhattan plots of CpG positons on chromosomes (**A**) Manhattan plot of CpG sites on all chromosomes. The horizontal axis is positions of chromosome, and the vertical axis is −log10 of *P*-value. The blue line and the red line are thresholds of 1e-8 and 1e-10, respectively. (**B**–**G**) Manhattan plots of CpG sites on chromosome 1, 2, 5, 10, 15, and 22, respectively.

Samples within different groups based on COG showed significantly different survival time according to previous analysis. To further study the relationship between DNA methylation and COG, we used CpGassoc package to perform EWAS. *P*-value was used to measure the relationship between the CpG sites methylation levels and tumor COG. The smaller the *P*-value, the closer the relationship between them. We screened 81 CpG sites with *P*<1e-8 as the threshold, and 13 CpG sites with *P*<1e-10 as the threshold. As shown in Figure 2A, the 13 CpG sites were distributed on chromosomes 1, 2, 5, 10, 15, and 22. For details, Figure 2B–G were enlarged Manhattan plots of CpG positions on chromosomes 1, 2, 5, 10, 15, and 22, respectively.

### Construction and evaluation of the logistic regression model in predicting COG risk group

**Figure 3:**
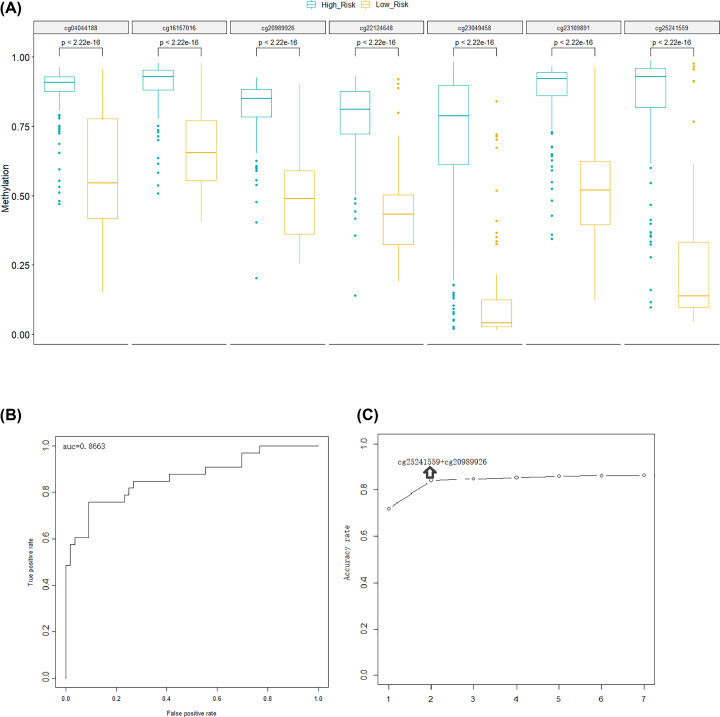
CpG-based model could well predict COG risk level in NB (**A**) Methylation levels of the seven CpG sites in the high-risk group and low-risk group. Blue: high-risk group; Yellow: low-risk group; Vertical axis: methylation level. (**B**) Prediction of the half of the 236 samples using the logistic regression model. (**C**) Evaluation of the logistic regression model in predicting COG risk level. The horizontal axis represents the number of included points, and the vertical axis represents the prediction accuracy.

Six of the 13 CpG sites were not annotated to any gene, and seven of them were found with corresponding genes, which were cg25241559 (SNED1), cg20989926 (RLBP1), cg22124648 (FGR), cg23109891 (SOX10), cg23049458 (L1TD1), cg04044188 (PDSS1), and cg16157016 (PICK1). As shown in Figure 3A, we analyzed the methylation levels of these seven sites between high COG and low COG sample group. *P*-values calculated by Wilcox method were all less than 0.05, indicating that the methylation levels of these seven CpG sites were significantly different between the high and low COG groups, and the methylation level in the high COG group was significantly higher than that in the low COG group. A logistic regression model based on the methylation levels of these seven CpG sites in a random divided training set with half of all samples was constructed to determine the COG risk groups. To verify the accuracy of this model, we used the other half of the samples for prediction. As shown in Figure 3B, the inflection points of the curve were all close to the upper left and the area under curve (AUC) was greater than 0.8 (AUC = 0.8663), indicating the high accuracy of the model. Using information-gain-based approach to sort the reliability of the seven CpG sites in predicting COG risk group from high to low, we found that the prediction accuracy of cg25241559 + cg20989926 reached 0.84, while the accuracy of the next points did not significantly increase, indicating that the model constructed by these two CpG sites was accurate enough for COG group prediction (Figure 3C).

## Discussion

The risk stratification based on clinical and biologic factors of patients with tumor has been used to determine the appropriate treatment and is criteria for predicting prognosis for decades. Since 2006, the COG has been collecting INRG data and revising the COG risk classification system to achieve better predictive performance [10]. However, patients with same risk stratification may have different clinical courses after receiving same therapy [11]. On the basis of the assumption that the occurrence and development of tumor are driven by genetic and biological characteristics, researchers have identified a large number of potential prognostic markers and established classification for improving treatment strategies [12–14]. Although several well-characterized genetic abnormalities, including DNA content [15], gain of chromosome arm 17q, and deletion of chromosome arm 1p and 11q have been discovered to be associated with outcome prediction in NB [16], features of these molecules are inadequate to explain the clinical heterogeneity [17].

There is increasing evidence that abnormal DNA methylation is highly related to the development and progression of many cancers including NB. Hypermethylation was found in aggressive tumors compared with low-grade ones [18], and the tumorigenic properties of NB was inhibited by reversing epigenetic changes in CpG island with demethylating agent [19]. Capper et al. reported that DNA methylation patterns might be useful for the accurate classification of olfactory NB [20]. In this study, we analyzed the methylation data of NB in TARGET database, and found that the DNA methylation in the whole-genome that was associated with MKI, an indicator of NB histological classification [7], was significantly related to prognosis and survival. Meanwhile, COG risk group significantly affected the prognosis and survival, also suggesting that the methylation characteristics showed good potential in NB prognosis prediction.

Furthermore, seven CpG sites with significantly different methylation levels in the high- and low-COG risk groups were identified, including cg25241559, cg20989926, cg22124648, cg23109891, cg23049458, cg04044188 and cg16157016, and logistic regression model with these CpG sites was constructed to accurately determine the COG risk of the samples. In addition, by analyzing the prediction reliability of these CpG sites, we found that the model constructed using cg25241559 and cg20989926 was accurate enough for COG risk group prediction. cg25241559 is a probe representing Sushi Nidogen and EGF-like domains 1 (SNED1), which encodes a secreted protein composed of characteristic domains commonly found in ECM proteins [21]. The crucial role of SNED1 in the development of several tumors has been discovered. Longati et al. identified SNED1 as a stromal marker that induces cisplatin resistance in head and neck squamous carcinoma [22]. Naba et al. reported SNED1 as characteristic protein of highly metastatic in mammary carcinoma, and the overexpression of SNED1 was associated with poor outcome for patients with ER−/PR− breast cancer [23]. The homozygous deletions of SNED1 might be involved in tumor metastasis of NB [24], while DNA methylation change of SNED1 was associated with the clinical course of NB [25]. However, the mechanisms of SNED1 involved in invasive phenotype remains unclear. Cg20989926 is a probe representing RLBP1, a retinaldehyde-binding protein gene that encodes human cellular retinaldehyde-binding protein [26]. Mutation of RLBP1 is associated with several inherited retinal disorders, such as Bothnia dystrophy [27], newfoundland rod-cone dystrophy [28], retinitis pigmentosa [29], and retinitis punctata albescens [30]. In oral cancer, the over expression of RLBP1 is associated with increased glucose uptake and aerobic glycolysis-mediated ATP synthesis [31]. The hypermethylation of RLBP1 is also recognized as one of the features of high-risk NB [25]. On the other hand, 5-hydroxymethylcytosine (5-hmC), a key component of DNA methylation, was proved to be related to hypoxic response of NB cell lines [32]. In another study on prognostic factors of NB using COG cohorts, 5-hmC profiles could be used as a biomarker for NB in children [33]. Perhaps the transcriptional network essential for promoting different NB phenotypes is regulated by distinct pattern of DNA modification, which requires further research in the future.

## Conclusion

With the discovery of key somatic and germline genomic alterations, new tumor classifications relying on molecular profiles of tumor and host are emerging to provide evidences for treatment strategy of NB. In the present study, the methylation data of NB from TARGET database were analyzed to investigate the relationship between DNA methylation and COG risk groups. Seven CpG sites that significantly associated with risk classification were identified, and the combination of SNED1 and RLBP1 were accurate enough for risk stratification. The present study provided a basis for the refinements in risk classification, although additional research will be needed to clarify the biological functions of these methylation variations to achieve higher accuracy.

## Abbreviations

AUC: area under curve
COG: Children’s Oncology Group
CpG: CpG island
ECM: extracellular matrix
EGF: Epidermal Growth Factor
EWAS: epigenome-wide association study
HR: hazard rate
INRG: International Neuroblastoma Risk Group
INSS: International Neuroblastoma staging system
MKI: mitosis-karyorrhexis index
MYCN: MYCN proto-oncogene, bHLH transcription factor
NB: neuroblastoma
SNED1: Sushi Nidogen and EGF-like domains 1
TARGET: Therapeutically Applicable Research to Generate Effective Treatments
5-hmC: 5-hydroxymethylcytosine
